# Extracellular Vesicles for Corneal Endothelial Repair

**DOI:** 10.3390/ijms27198789

**Published:** 2026-09-30

**Authors:** Ana Kolenc, Elvira Maličev, Zala Lužnik Marzidovšek

**Affiliations:** 1 Blood Transfusion Centre of Slovenia, 1000 Ljubljana, Slovenia; ana_kolenc@ztm.si (A.K.); elvira.malicev@ztm.si (E.M.); 2 Eye Hospital, University Medical Centre Ljubljana, 1000 Ljubljana, Slovenia; 3 Faculty of Medicine, University of Ljubljana, 1000 Ljubljana, Slovenia

**Keywords:** mesenchymal stem cells, extracellular vesicles, corneal repair, corneal endothelium, regenerative medicine

## Abstract

The corneal endothelium is essential for maintaining corneal transparency and visual function through regulation of stromal hydration. However, human corneal endothelial cells have limited regenerative capacity in vivo, and their loss due to aging, trauma, surgery, or diseases such as Fuchs endothelial corneal dystrophy can lead to corneal oedema and visual impairment. Although corneal transplantation remains the primary treatment for endothelial dysfunction or loss, donor tissue shortages and postoperative complications have driven the search for alternative therapies. Extracellular vesicles (EVs) have emerged as promising cell-free therapeutic agents that mediate intercellular communication through the transfer of bioactive molecules. Recent studies have investigated two distinct EV sources in corneal endothelial biology: mesenchymal-stem-cell-derived EVs, which have demonstrated cytoprotective and regenerative effects, and corneal endothelial cell-derived EVs, which appear to modulate endothelial homeostasis and, depending on their cargo, may either limit proliferation or exert protective effects. This review summarizes current evidence on EV biology, their distinct cellular sources, and their potential roles in corneal endothelial repair.

## 1. Introduction

The corneal endothelium is a specialised monolayer of cells that is essential for maintaining corneal transparency and visual function by regulating stromal hydration. This function occurs through the classical pump–leak mechanism, in which the endothelial monolayer acts as a semipermeable barrier that permits passive influx of aqueous humour and solutes while actively transporting ions to remove excess fluid from the stroma into the anterior chamber, preserving corneal deturgescence [1]. Human corneal endothelial cells (CEnCs) form a single hexagonal monolayer on the posterior surface of the cornea, immediately anterior to Descemet’s membrane. Unlike many other cell types, CEnCs exhibit limited proliferative capacity in vivo because they are arrested in the G1 phase of the cell cycle. Although CEnCs remain quiescent in vivo, they stay functionally active and continuously perform barrier and ion-transport functions that help maintain stromal deturgescence. Consequently, physiological ageing and pathological injury lead to a progressive decline in endothelial cell density, with cell loss compensated primarily by cellular enlargement and migration rather than proliferation [2]. When the endothelial cell population falls below a critical threshold, the remaining cells can no longer maintain stromal deturgescence, leading to corneal oedema and loss of transparency with progressive visual impairment. Endothelial decompensation is characteristic of disorders such as Fuchs endothelial corneal dystrophy (FECD) and bullous keratopathy, major causes of corneal endothelial failure and vision loss in Western countries [3].

Current management of corneal endothelial diseases still relies primarily on endothelial keratoplasty, with Descemet Membrane Endothelial Keratoplasty (DMEK) and Descemet Stripping Automated Endothelial Keratoplasty (DSAEK) as the surgical gold standard for restoring endothelial function. Nevertheless, these procedures remain dependent on the availability of suitable donor corneas. They are associated with high procedural costs, technically demanding surgery, graft failure or rejection, and the need for prolonged postoperative follow-up and immunosuppressive therapy in selected patients [4,5]. These limitations have driven the search for alternative regenerative approaches that restore corneal endothelial function while reducing reliance on donor tissue.

Regenerative medicine offers new therapeutic strategies, including cell-based therapies, tissue engineering, biomaterials, and gene-based approaches. Among the most advanced approaches is the intra-cameral injection of cultured human corneal endothelial cells combined with a Rho-associated protein kinase (ROCK) inhibitor, which has demonstrated encouraging clinical outcomes and represents one of the first successful cell-based therapies for corneal endothelial dysfunction [6]. In parallel, stem cell-based therapies have gained considerable attention because they can differentiate into multiple cell lineages and, more importantly, promote tissue repair by secreting immunomodulatory, anti-inflammatory, and regenerative factors. Despite their therapeutic potential, cell-based approaches still face important challenges, including limited cell survival and engraftment following transplantation, potential immunogenicity, stringent regulatory requirements, and the logistical complexities associated with large-scale manufacturing, storage, and distribution [7].

To overcome the limitations of cell-based therapies while preserving much of their therapeutic potential, extracellular vesicles (EVs) have emerged as a promising cell-free strategy for corneal endothelial repair and regeneration [8]. Even though the evidence is still preclinical and largely based on in vitro conditions, it is coherent. EVs are lipid bilayer-enclosed nano- to micrometre-sized particles secreted by all cell types under both physiological and pathological conditions. They serve as key mediators of intercellular communication by transferring a diverse biomolecular cargo, including proteins, lipids, mRNAs, miRNAs, and other regulatory molecules [9], thereby modulating cellular proliferation, migration, survival, immunomodulation, and tissue regeneration [10,11,12]. Compared with cell-based therapies, EVs offer several important advantages, including lower immunogenicity, without risk of uncontrolled cellular proliferation or tumorigenesis, and greater potential for standardised manufacturing [13]. Furthermore, their natural ability to deliver bioactive molecules to target cells, together with the potential to bioengineer their cargo or surface properties, has positioned EVs as a versatile platform for next-generation regenerative therapies [8,14,15].

Unlike our previous review [8], which broadly addressed EVs in corneal wound and ocular surface regeneration, this review summarises current knowledge on the therapeutic potential of EVs for corneal endothelial repair, with particular emphasis on their biological properties, cellular sources, mechanisms of action, and the challenges that must be addressed for successful clinical translation.

## 2. Extracellular Vesicles: Biogenesis, Classification, and Biological Functions

Understanding the biogenesis, cargo composition, and biological functions of EVs is essential for elucidating their potential role in corneal endothelial regeneration. EVs are membrane-bound particles released by virtually all cell types into the extracellular environment. Initially considered cellular debris, EVs are now recognised as important mediators of intercellular communication involved in numerous physiological and pathological processes. They transport a diverse cargo of proteins, lipids, nucleic acids, and metabolites, influencing recipient cell behaviour [16,17,18]. Research over the past two decades has revealed that EVs play important roles in immune regulation, tissue repair, angiogenesis, and disease progression, particularly in cancer, neurodegenerative disorders, and cardiovascular diseases [19,20]. Kamra et al. [21] recently provided a broader overview of EV biology, including cargo composition and therapeutic mechanisms.

**Figure 1:**
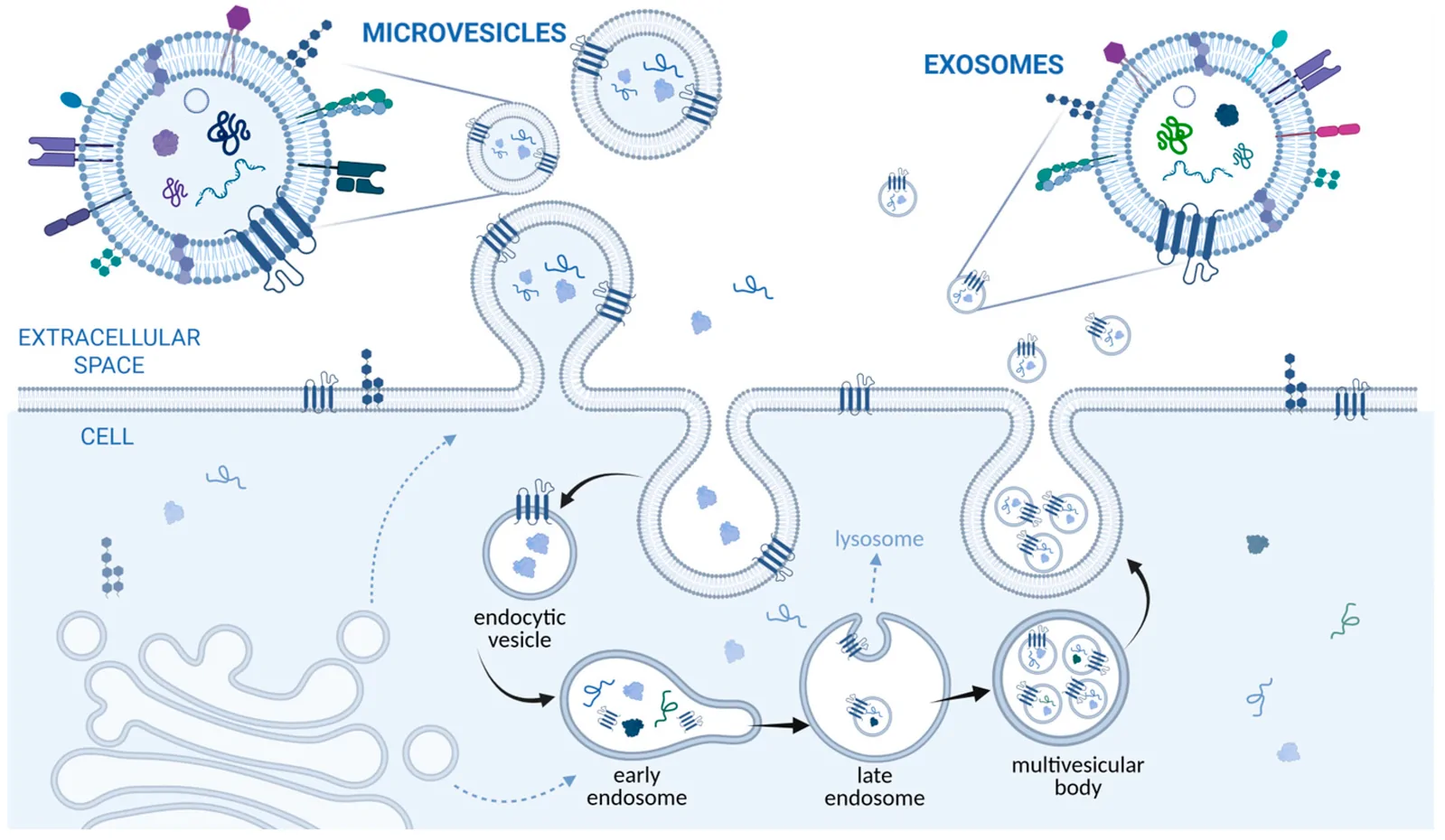
Biogenesis of ectosomes/microvesicles and exosomes. They represent two major classes of extracellular vesicles (EVs) and differ in their size ranges. According to Jeppesen et al. (2023) [26], exosomes released from multivesicular bodies are approximately 30–120 nm in diameter, whereas ectosomes (the various classes of plasma membrane-budding vesicles) range from approximately 30 nm to 10 µm. Apoptotic bodies (not depicted in the figure) represent one of the largest classes of EVs and can reach up to approximately 5 µm in diameter. In addition to these categories, an increasing number of distinct EV populations and extracellular particles are being described, highlighting EV heterogeneity and complexity. Adapted and summarised from MISEV2023 and Jeppesen et al. (2023) [9,26]. Created with BioRender. Jazbec Gradišar, K. (2026) https://BioRender.com/x72uyiy.

EVs are generated through distinct cellular pathways, leading to different EV subtypes. The classical biogenesis classification includes exosomes, microvesicles or ectosomes (Figure 1) and apoptotic bodies. Exosomes originate from the endosomal pathway. Their formation begins with inward budding of the plasma membrane, generating early endosomes. These mature into late endosomes, where intraluminal vesicles (ILVs) are formed through inward budding of the endosomal membrane. The resulting structures, known as multivesicular bodies (MVBs), can either fuse with lysosomes for degradation or merge with the plasma membrane, releasing ILVs as exosomes into the extracellular space [16,22]. The Endosomal Sorting Complex Required for Transport (ESCRT) machinery regulates ILV formation, as do ESCRT-independent mechanisms involving lipids such as ceramide and proteins including tetraspanins [17,23,24]. Microvesicles are formed through direct outward budding and fission of the plasma membrane [9,25], while apoptotic bodies arise from apoptotic cell fragmentation. Although these categories remain widely used to describe EV populations, current consensus recommendations (MISEV2023) emphasise that biogenesis-based terminology should be used cautiously, as EV isolation methods generally do not allow isolation of individual populations. The EV field has also expanded to encompass diverse EV populations, including migrasomes, exophers, autophagy-related EVs, mitovesicles, matrix vesicles, and large oncosomes, as well as non-vesicular extracellular particles such as exomeres and supermeres [9,26].

EVs primarily transfer bioactive molecules between cells. Through their cargo, EVs can alter gene expression, metabolic activity, and signalling pathways in recipient cells. This communication occurs locally within tissues and systemically through body fluids [18]. EVs contribute significantly to immune system modulation. Antigen-presenting cells release EVs carrying major histocompatibility complex (MHC) molecules that can stimulate T-cell responses. Conversely, tumour-derived EVs may suppress immune activity and facilitate immune evasion [27]. Stem-cell-derived EVs have demonstrated regenerative potential in various tissues. They promote angiogenesis, reduce inflammation, and stimulate cellular proliferation by delivering growth factors, microRNAs, and signalling proteins. These properties make EVs promising candidates for cell-free regenerative therapies [28], as well as potential therapeutic agents in tissue repair, including corneal endothelial regeneration.

Extracellular vesicles represent a rapidly evolving field of biomedical research. Their diverse biogenesis pathways, heterogeneous populations, and multifunctional biological activities position them as central regulators of intercellular communication. Given this review’s focus on corneal endothelial repair, understanding these fundamental EV properties provides an important framework for interpreting their therapeutic potential in this tissue context.

## 3. Sources of EVs Relevant for Corneal Endothelial Repair

EVs from different cell sources may exhibit distinct cargo profiles and biological effects that could influence their potential for corneal endothelial repair. Current evidence includes EVs from mesenchymal stem cells, corneal cells, induced pluripotent stem cells, and blood cells, with effects ranging from cytoprotection and regenerative activity to regulation of endothelial homeostasis and quiescence.

### 3.1. Mesenchymal Stem Cell-Derived EVs

Bone-marrow-derived mesenchymal stem cells (BM-MSCs) are among the most extensively studied sources of therapeutic EVs. BM-MSC-derived EVs contain growth factors, cytokines, and regulatory miRNAs that can promote cell survival, modulate inflammation, and support tissue repair [29]. In ocular research, BM-MSC-derived EVs have shown cytoprotective, anti-inflammatory, regenerative, and neuroprotective effects, including reduced oxidative stress and apoptosis, enhanced wound healing and re-epithelialisation, reduced inflammation in acute ocular surface injury and dry eye disease models, and promotion of corneal nerve regeneration [30,31,32]. MSC-derived EVs have been shown to activate signalling pathways such as PI3K/Akt and ERK1/2, which are associated with cell survival and regenerative responses [33,34]. Specifically, in in vitro studies with hCEnC, BM-MSC-derived EVs attenuated endothelial apoptosis, activated cellular migration and stimulated regenerative repair pathways [11,35]. Adipose tissue provides an abundant and accessible source of MSCs. Adipose-derived MSCs (AT-MSCs) secrete EVs enriched in regenerative growth factors and anti-inflammatory miRNAs. Compared with bone-marrow-derived MSCs, AT-MSCs can be obtained through minimally invasive procedures [36]. Studies have shown that AT-MSC-derived EVs promote epithelial and stromal wound healing, support metabolic homeostasis, and protect against apoptosis [37,38,39]. They may also support endothelial repair by modulating inflammatory responses and enhancing cell survival. Their effects have been associated with the transfer of miRNAs involved in cell-cycle regulation and tissue repair. Human umbilical-cord-derived MSCs (UC-MSCs) have also gained considerable attention because of their high proliferative capacity and relatively low immunogenicity. EVs isolated from UC-MSCs contain regenerative and anti-inflammatory molecules, including regulatory miRNAs [40]. Experimental studies have shown that their exosomes can promote corneal epithelial cell proliferation and migration and enhance corneal wound healing, supporting their potential for ocular tissue regeneration [41]. These properties may be particularly relevant to conditions in which inflammation and cellular stress contribute to corneal endothelial damage.

Although bone marrow, umbilical cord, and adipose tissue are the most frequently reported sources of MSCs for EV isolation in both preclinical and clinical studies, MSCs derived from other tissues have also been investigated. These include dental pulp, endometrium, synovium, menstrual blood, hair follicles, kidney, and dermal tissue, highlighting the broad range of potential MSC sources for therapeutic EV production. However, despite their reported regenerative and therapeutic potential, MSC-derived EVs have rarely been shown to support corneal endothelial regeneration [42,43].

### 3.2. Corneal-Cell-Derived EVs

Corneal stromal stem cells (CSSCs) are resident progenitor cells located within the limbal stroma. EVs secreted by CSSCs have demonstrated regenerative and immunomodulatory effects in experimental models of corneal injury, including reduced inflammation and fibrosis and enhanced tissue repair [44,45]. Because CSSCs originate from ocular tissue, their EVs may have greater tissue specificity than EVs derived from non-ocular stem cells. This characteristic could facilitate targeted modulation of the corneal microenvironment and support endothelial recovery. However, to the best of our knowledge, their potential role in promoting corneal endothelial cell survival, regeneration, or functional recovery has not yet been directly investigated.

**Figure 2:**
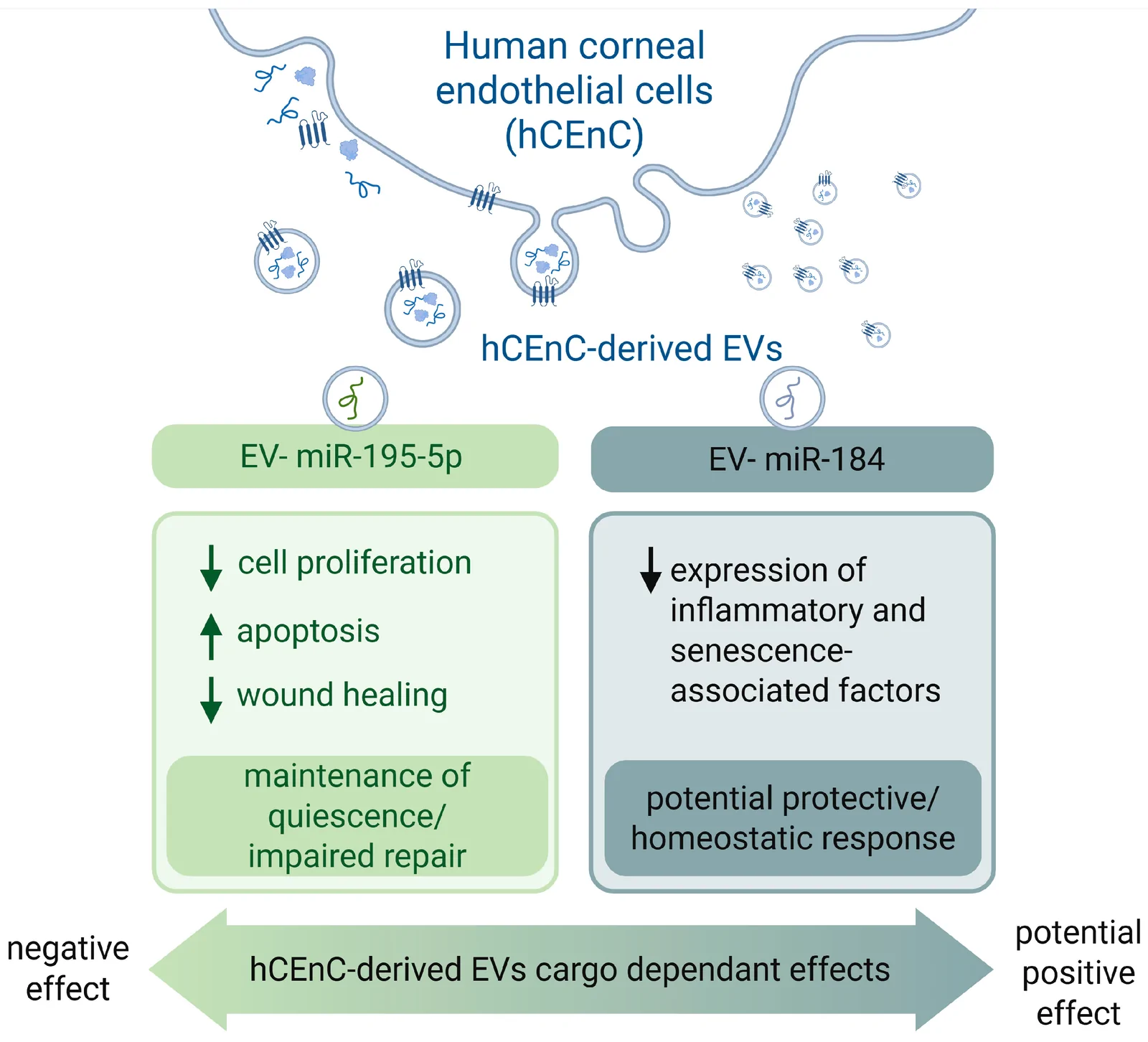
Contrasting effects of CEnC-derived EV cargo. Summarised from [46,47]. Created with BioRender. Jazbec Gradišar, K. (2026) https://BioRender.com/hxyafia.

CEnCs are another potential source of EVs. In contrast to MSC-derived EVs, CEnC-derived EVs may have distinct effects on endothelial cell behaviour and homeostasis. Early studies showed that neighbouring endothelial cells internalise CEnC-derived EVs, which reduce proliferation, increase apoptosis, and impair endothelial wound healing, suggesting a role in maintaining CEnC quiescence rather than promoting regeneration [46]. Sequencing of these endothelial EVs identified 13 miRNAs linked to cell-cycle arrest, adherens junctions, and p53 signalling, including EV-enriched miR-195-5p, which negatively regulates CEnC proliferation. Inhibiting miR-195-5p enhanced proliferation, viability, and wound healing while reducing apoptosis [46]. In contrast, Yamashita et al. identified miR-184 as a potentially protective EV-associated miRNA in CEnCs. Increased release of EV-associated miR-184 under oxidative stress was proposed as a compensatory mechanism, while miR-184 overexpression suppressed the expression of inflammatory and senescence-associated factors, including VEGF, IL-6, MCP-1, and MMPs [47]. Together, these findings highlight that the biological effects of CEnC-derived EVs are cargo-dependent and may range from maintaining endothelial quiescence to supporting protective responses to cellular stress (Figure 2). However, the limited availability of human CEnCs remains a major challenge for their use as a scalable EV source. The widest gap is the near-total absence of in vivo functional repair studies for endogenous corneal cell-derived EVs.

Another potential application is the engineering of CEnC-derived EVs for targeted delivery of therapeutic miRNAs. Lee et al. demonstrated the feasibility of using these EVs as delivery vehicles to the mouse corneal endothelium in vivo [48].

### 3.3. Induced Pluripotent Stem Cell-Derived EVs

Induced pluripotent stem cells (iPSCs) represent a potentially scalable source of EVs with regenerative properties. iPSC-derived EVs contain regulatory miRNAs, proteins, and other bioactive molecules that may support cell survival, proliferation, and tissue repair [49,50,51]. In ocular research, iPSC-derived corneal endothelial-like cells have demonstrated regenerative potential in animal models of endothelial dysfunction, and early clinical studies have begun to evaluate their therapeutic application [52,53].

Wang et al. demonstrated that both iPSC- and MSC-derived EVs accelerated corneal epithelial defect healing, with iPSC-derived EVs showing a stronger effect. This was associated with upregulation of cyclin A and CDK2, promoting corneal epithelial cells to transition from the G0/G1 to the S phase of the cell cycle [54]. Whether iPSC-derived EVs can similarly promote corneal endothelial repair remains largely unexplored. However, evidence from other endothelial tissues supports their potential regenerative effects. In human coronary artery endothelial cells, miR-126-enriched iPSC-derived EVs enhanced cellular functions relevant to ischemic tissue repair, including proliferation, metabolic activity, survival, migration, and angiogenic capacity [55]. They further supported these effects in a murine model of acute limb ischemia. Beyond cardiovascular applications, iPSC-derived EVs have also shown beneficial effects in models of acute kidney injury [56], retinal ganglion [57], diabetic wound healing [58], and periodontal bone defects [59].

### 3.4. Other Cell Sources

Additional EV sources have also shown therapeutic potential in corneal endothelial repair. Beyond the corneal endothelium, platelet-derived EVs have also shown therapeutic potential in an alkali-burn mouse model, where platelet-derived EVs loaded with kaempferol reduced corneal neovascularisation [60].

In a study comparing MSC-derived EVs with serum-derived EVs, both showed pro-angiogenic activity; however, serum-derived EVs showed a weaker protective effect against apoptosis of hCEnC in vitro than MSC-derived EVs [35]. This suggests that the biological effects of EVs may vary substantially by source and, consequently, by cargo.

Human amniotic epithelial-cell-derived EVs represent another potential source for corneal repair. Researchers have investigated their effects primarily in corneal epithelial injury, where they have been associated with enhanced epithelial regeneration, reduced scar formation, and restored corneal transparency [61]. However, their potential for direct corneal endothelial regeneration remains to be further explored.

## 4. Mechanisms of EV-Mediated Corneal Endothelial Repair

EVs can modulate corneal endothelial cell function by transferring proteins, lipids, mRNAs, miRNAs, and other bioactive molecules. After recipient cells take up EV cargo, it can influence signalling pathways involved in cell survival, apoptosis, proliferation, inflammation, oxidative stress, and tissue repair [19]. The specific effects depend on the cellular source and molecular cargo of the EVs (Table 1). The following sections summarise the main mechanisms implicated in EV-mediated modulation of corneal endothelial repair. Although these mechanisms are well described in various cell types, some of their roles in corneal endothelial cells remain incompletely defined, as discussed below.

### 4.1. Cellular Uptake and Intracellular Signalling

EVs begin their biological effects by interacting with recipient cells. EVs can be internalised through several mechanisms, including endocytosis, phagocytosis, micropinocytosis, and, in some contexts, direct membrane fusion. Following internalisation, EV cargo may interact with intracellular signalling networks and influence gene expression [25]. EV-associated miRNAs and proteins have been implicated in modulating pathways involved in cell survival, proliferation, and tissue repair. Among these, the phosphoinositide 3-kinase/protein kinase B (PI3K/Akt), extracellular signal-regulated kinase (ERK1/2), and Wnt/β-catenin pathways have been implicated in cellular survival and regenerative responses in various cell types [34]. However, their relevance to EV-mediated effects in corneal endothelial cells remains unclear.

### 4.2. Protection Against Oxidative Stress and Apoptosis

Oxidative stress contributes significantly to corneal endothelial dysfunction, particularly in FECD, where excessive reactive oxygen species (ROS) generation can promote mitochondrial dysfunction, cellular senescence, DNA damage, and apoptosis [62]. EVs may exert cytoprotective effects by delivering regulatory molecules that modulate cellular stress responses and promote endothelial cell survival. In human corneal endothelial cells, MSC-derived EVs have been shown to protect against endoplasmic reticulum stress-induced apoptosis by suppressing activation of the PERK/eIF2α/ATF4/CHOP pathway, thereby improving cell survival under stress conditions [35]. Similarly, MSC-derived EVs reduced apoptosis in serum deprivation-injured human corneal endothelial cells while enhancing proliferation and migration, supporting their cytoprotective effects [11]. Furthermore, adipose-derived MSC exosomes attenuated H_2_O_2_-induced oxidative stress and senescence in human corneal endothelial cells, suppressed endothelial–mesenchymal transition, and promoted regenerative responses [63]. Beyond exogenous EVs, endogenous EV release may also contribute to corneal endothelial homeostasis. EV-associated miR-184 released by human corneal endothelial cells has been implicated in limiting endothelial degeneration under oxidative stress [47]. Similarly, in FECD, inhibition of the EV biogenesis pathway has been associated with impaired mitochondrial function and corneal endothelial cell death, further suggesting a role for endogenous EVs in maintaining endothelial homeostasis [64]. Collectively, these findings support a cytoprotective role of EVs in corneal endothelial cells, particularly under conditions of cellular stress.

### 4.3. Promotion of Endothelial Repair Through Proliferation and Migration

Although mature human corneal endothelial cells have traditionally been considered to have limited proliferative capacity in vivo, their residual proliferative and migratory responses may be stimulated under appropriate conditions. EVs contain bioactive molecules, including growth factors and regulatory RNAs, that can modulate pathways involved in cell-cycle regulation, tissue repair, and cellular homeostasis. In human corneal endothelial cells, adipose MSC-derived exosomes have been reported to promote cell proliferation and regenerative responses while reducing stress-induced senescence [63]. In addition, MSC-derived EVs can enhance migration and wound closure in human corneal endothelial cells, potentially restoring endothelial continuity after injury [11]. EVs isolated from human platelet concentrates have been shown to increase the proliferation, migration, and adhesion of corneal endothelial cells in vitro [12]. Importantly, EV effects appear to depend on their cellular origin and molecular cargo. Human corneal endothelial cell-derived EVs have been reported to reduce proliferation, increase apoptosis, and impair wound-healing responses [46]. These findings highlight the importance of EV source and cargo characterisation when developing EV-based approaches for corneal endothelial dysfunction.

### 4.4. Restoration of Endothelial Barrier and Pump Function

An important goal of corneal endothelial repair is restoring endothelial barrier integrity and pump function. EVs may contribute by delivering bioactive molecules that regulate cell survival, adhesion, organisation, and functional recovery. MSC-derived EVs have been shown to promote repair of damaged human corneal endothelial cells by enhancing survival, proliferation, and migration, suggesting a potential role in restoring endothelial monolayer integrity [11,63]. In addition, platelet-derived EVs have been reported to maintain the expression of corneal endothelial markers, including ZO-1, N-cadherin, and Na^+^/K^+^-ATPase, in cultured corneal endothelial cells [12]. However, direct evidence demonstrating EV-mediated restoration of endothelial barrier function and pump activity remains limited. Further studies are therefore needed to determine whether the observed effects on endothelial phenotype and cellular repair translate into restoration of functional barrier and pump activity, using objective functional endpoints such as transendothelial electrical resistance, Na^+^/K^+^-ATPase activity, expression and organisation of tight-junction proteins such as ZO-1 and claudins, and pump density.

### 4.5. Immunomodulatory and Anti-Inflammatory Effects

Inflammation plays an important role in corneal endothelial injury and postoperative complications following corneal transplantation. Pro-inflammatory cytokines such as tumour necrosis factor-α (TNF-α), interleukin-1β (IL-1β), and interleukin-6 (IL-6) can impair endothelial cell function and accelerate cell loss. EVs have immunomodulatory properties that may help establish a regenerative microenvironment. Adipose MSC-derived exosomes promoted corneal endothelial wound healing and migration in vitro and in vivo, while also inhibiting transforming growth factor-β (TGF-β)-associated epithelial–mesenchymal transition and cellular senescence [63]. Importantly, beyond these direct effects on corneal endothelial cells, broader MSC-EV research shows that MSC-derived EVs can suppress inflammatory responses while promoting anti-inflammatory effects, including increased interleukin-10 (IL-10) and TGF-β production and expansion of regulatory T cells [11,63]. EVs can also modulate immune cell behaviour by promoting alternative macrophage activation and polarisation towards an anti-inflammatory M2 phenotype, thereby reducing tissue damage and supporting repair processes [29]. However, whether these immunomodulatory mechanisms contribute directly to EV-mediated corneal endothelial repair remains to be established.

## 5. Future Directions

EVs derived from MSCs, corneal-derived cells, and iPSCs have emerged as promising candidates for corneal endothelial repair. Their ability to modulate inflammation, reduce oxidative stress and apoptosis, and promote cellular repair highlights their potential as cell-free therapies for endothelial dysfunction. Despite these encouraging advances, several scientific, technical, and regulatory challenges remain before EV-based therapies can be successfully translated into routine clinical practice.

### 5.1. Standardisation, Manufacturing, and Regulatory Challenges

Standardisation of EV isolation, purification, characterisation, and quality-control protocols remains essential, as different isolation methods produce vesicles with variable purity and biological activity [65]. In parallel, scalable Good Manufacturing Practice (GMP)-compliant production, batch-to-batch consistency, storage stability, and comprehensive evaluation of biodistribution, immunogenicity, toxicity, and long-term safety will be required to meet regulatory standards and enable clinical implementation [66].

### 5.2. Identification and Engineering of Therapeutic EV Cargo

Although numerous studies have demonstrated the regenerative potential of EVs, the specific bioactive molecules responsible for their therapeutic effects remain incompletely understood. Future studies integrating transcriptomic, proteomic, and metabolomic analyses are needed to identify the key proteins, lipids, and regulatory RNAs that mediate corneal endothelial regeneration [19]. Advances in EV bioengineering may further enhance therapeutic efficacy by enriching EVs with regenerative miRNAs, proteins, growth factors, or gene-editing components, thereby improving targeting efficiency and enabling personalised treatment strategies [67].

### 5.3. Advanced Delivery Strategies

Efficient delivery remains a major challenge for ocular EV therapies. Future research should optimise intra-cameral administration and investigate sustained-release platforms, including hydrogels, nanofibres, contact lenses, and biodegradable scaffolds, to improve EV retention and therapeutic efficacy. Beyond their intrinsic regenerative properties, EVs may also serve as targeted delivery vehicles. A proof-of-concept study demonstrated that human corneal endothelial cell-derived small EVs successfully delivered peptide cargo to the mouse corneal endothelium following intra-cameral injection, highlighting their potential for targeted therapeutic delivery [48]. Combining EVs with biomaterial-based tissue engineering approaches may further enhance corneal endothelial regeneration and expand their application in donor cornea preservation and eye banking.

### 5.4. Eye-Banking

Beyond their potential for regenerative treatment of corneal endothelial dysfunction, EVs may also have applications in eye banking by protecting corneal endothelial cells during donor cornea storage and thereby preserving endothelial viability and function before transplantation. Our preliminary data suggest that umbilical cord MSC-derived EVs may exert a cytoprotective effect on human donor corneal endothelium under inflammatory stress in Eusol-C preservation medium. However, further studies are needed to determine the optimal EV concentration, timing and duration of exposure, and compatibility with preservation media such as Eusol-C, Optisol-GS, and Life4 °C, as well as their effects on endothelial viability and function during storage.

### 5.5. Safety, Immunogenicity, and Biodistribution

Despite the generally favourable safety profile reported for MSC-derived EVs in preclinical studies, potential adverse effects, immunogenicity, and off-target biodistribution require careful evaluation before clinical translation. The anterior chamber presents a unique microenvironment characterised by immune privilege and continuous aqueous humor turnover, which may influence EV retention, distribution, cellular uptake, and clearance following intracameral administration [9,68]. Although MSC-derived EVs are generally considered relatively low in immunogenicity, their biological effects may vary by cellular source, molecular cargo, production conditions, and route of administration [68]. Potential adverse effects, including unwanted modulation of local immune responses, off-target cellular uptake, and effects on non-target ocular tissues, should therefore be systematically investigated. Future studies should assess EV persistence and biodistribution within the anterior chamber and surrounding ocular tissues, as well as local and systemic immune responses, ocular toxicity, and long-term effects on corneal endothelial cells and other anterior segment structures [9,68]. These considerations will be particularly important for determining the optimal dose, administration frequency, and formulation of EV-based therapies for corneal endothelial diseases.

### 5.6. Clinical Translation

**Figure 3:**
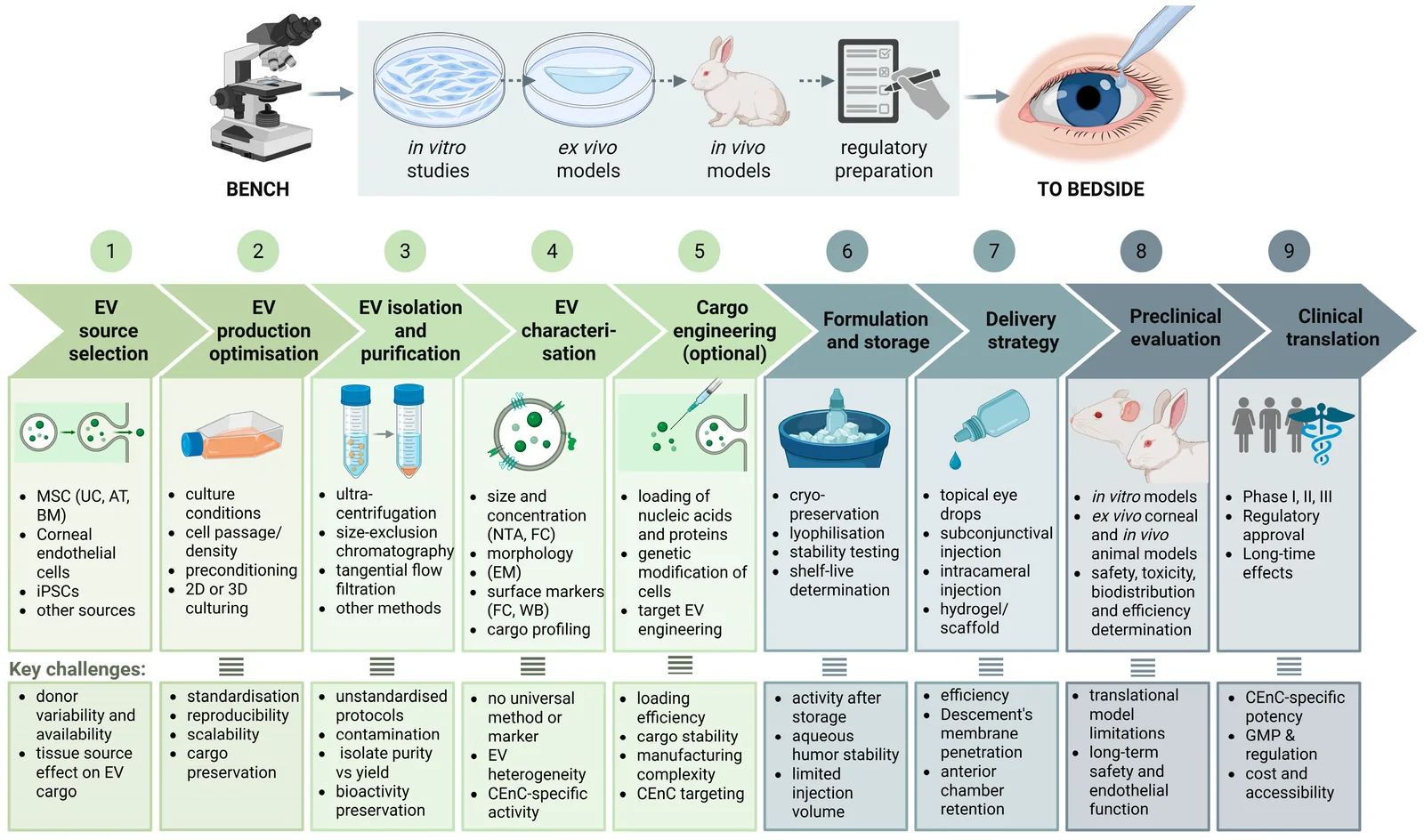
From bench to bedside: translational roadmap for extracellular-vesicle-based therapies for corneal endothelial repair. Overview of the key steps involved in the development of extracellular vesicle therapeutics, including source selection, production optimisation, isolation, characterisation, engineering, formulation, delivery, preclinical evaluation, and clinical translation, together with the major challenges associated with each stage. Adapted and summarised from [9,68,69,70]. Abbreviations: EV—extracellular vesicles; MSC—mesenchymal stem cells; UC—umbilical cord; AT—adipose tissue; BM—bone marrow; iPSCs—induced pluripotent stem cells; NTA—nanoparticle tracking analysis; FC—flow cytometry; EM—electron microscopy; WB—Western blotting; CEnCs—corneal endothelial cells; GMP—good manufacturing practice. Created with BioRender. Jazbec Gradišar, K. (2026) https://BioRender.com/kod4tcq.

Although preclinical studies have demonstrated encouraging regenerative effects, clinical evidence remains limited. Well-designed randomised clinical trials are needed to establish the efficacy, optimal dosing, treatment frequency, and long-term safety of EV-based therapies for corneal endothelial diseases (Figure 3). In the future, personalised approaches using autologous or induced pluripotent stem cell-derived EVs may offer opportunities to tailor EV characteristics and potentially reduce immunological concerns.

## 6. Conclusions

Corneal endothelial dysfunction remains an important cause of corneal vision loss, and treatment of established endothelial failure still relies largely on donor corneal transplantation. Preclinical evidence suggests that EVs may represent a promising cell-free therapeutic approach for corneal endothelial repair by modulating cellular survival, oxidative stress, inflammation, proliferation, and migration. However, important challenges remain before clinical translation.

Future research should prioritise (1) standardised, MISEV-compliant characterisation of EVs, including comprehensive proteomic, lipidomic, and RNA cargo profiling to identify the components responsible for their effects on corneal endothelial cells; (2) rigorous preclinical evaluation of dose–response relationships, biodistribution, functional endothelial recovery, and local and systemic safety using clinically relevant models; and (3) development of a translational framework for early-phase clinical studies, including GMP-compatible manufacturing, quality-control criteria, appropriate delivery and dosing strategies, and predefined safety endpoints for potential first-in-human trials. These steps will be essential to determine whether EV-based therapies can safely and meaningfully restore corneal endothelial function.

## Figures and Tables

**Table 1:** Summary of extracellular vesicle sources, cargo, mechanisms, and experimental evidence for corneal endothelial repair.

EV Cell Source	EV-Associated Cargo/Biomolecule Class	Principal Mechanisms Associated with EV-Mediated Effects	Study Model	Main Effect/Evidence
**MSC-derived EVs**				
BM-MSC-EVs	Growth factors, cytokines, regulatory miRNAs	PI3K/Akt, ERK1/2; modulation of oxidative stress and inflammation	hCEnCs in vitro	↑ cell survival; ↓ oxidative stress and apoptosis; ↑ wound healing
AT-MSC-EVs	Regenerative growth factors, anti-inflammatory miRNAs	Regulation of cell-cycle and tissue-repair pathways; suppression of oxidative stress and senescence	corneal in vitro models, evidence on endothelium is limited	↑ proliferation and regenerative responses; ↓ oxidative stress, senescence and endothelial–mesenchymal transition
UC-MSC-EVs	Regulatory miRNAs; regenerative and anti-inflammatory molecules	Pro-regenerative and anti-inflammatory signalling	corneal in vitro models, evidence on endothelium is limited	Corneal wound healing and epithelial repair; potential relevance to endothelial recovery
**Corneal cell-derived EVs**				
CSSC-EVs	Not yet fully characterised	Immunomodulatory and regenerative effects	Experimental corneal injury models	↓ inflammation and fibrosis; ↑ tissue repair; direct CEnC regenerative effects not yet demonstrated
CEnC-EVs	miR-195-5p	Cell-cycle regulation; adherens junction and p53-associated pathways	hCEnCs in vitro	↓ proliferation; ↑ apoptosis; ↓ wound healing; associated with endothelial quiescence
	miR-184	Regulation of inflammatory and senescence-associated factors		↓ VEGF, IL-6, MCP-1 and MMPs; potential protective/homeostatic response
**Other cell-derived EVs**				
iPSC-EVs	Regulatory miRNAs, proteins and other bioactive molecules	Pro-survival, proliferative and tissue-repair signalling	Primarily investigated in non-corneal tissues	Potential regenerative effects; direct evidence for corneal endothelial repair remains largely unexplored
Platelet-EVs	Growth factors (PDGF, EGF, TGF-β, HGF, IGF, VEGF)	Pro-regenerative effects; therapeutic cargo delivery	hCEnCs in vitro, mouse corneal neovascularisation model	↑ CEnC proliferation, migration and adhesion; ↓ corneal neovascularisation
Serum-EVs	Not yet fully characterised	Pro-angiogenic and cytoprotective effects	hCEnCs in vitro	Pro-angiogenic activity; weaker protection against apoptosis than MSC-derived EVs
Human amniotic epithelial cell-EVs	Not yet fully characterised	Extracellular matrix and tissue-repair modulation	Investigated in corneal epithelium.	↑ epithelial regeneration; ↓ scar formation; ↑ corneal transparency; direct CEnC effects remain to be explored

Abbreviations: EV—extracellular vesicles; MSC—mesenchymal stem cells; UC—umbilical cord; AT—adipose tissue; BM—bone marrow; hCEnCs—human corneal endothelial cells; CSSC—corneal stromal stem cells; VEGF—vascular endothelial growth factor; IL-6—interleukin-6; MCP-1—monocyte chemoattractant protein-1; MMPs—matrix metalloproteinases; PDGF—platelet-derived growth factor; EGF—epidermal growth factor; TGF-β—transforming growth factor-beta; HGF—hepatocyte growth factor; IGF—insulin-like growth factor.

## Data Availability

No new data were created or analyzed in this study. Data sharing is not applicable to this article.
